# Treadmill Workstations: The Effects of Walking while Working on Physical Activity and Work Performance

**DOI:** 10.1371/journal.pone.0088620

**Published:** 2014-02-20

**Authors:** Avner Ben-Ner, Darla J. Hamann, Gabriel Koepp, Chimnay U. Manohar, James Levine

**Affiliations:** 1 Carlson School of Management, University of Minnesota-Twin Cities, Minneapolis, Minnesota, United States of America; 2 School of Urban and Public Affairs, University of Texas-Arlington, Arlington, Texas, United States of America; 3 Obesity Solutions, Mayo Clinic and Arizona State University, Scottsdale, Arizona, United States of America; Pennington Biomedical Research Center, United States of America

## Abstract

We conducted a 12-month-long experiment in a financial services company to study how the availability of treadmill workstations affects employees’ physical activity and work performance. We enlisted sedentary volunteers, half of whom received treadmill workstations during the first two months of the study and the rest in the seventh month of the study. Participants could operate the treadmills at speeds of 0–2 mph and could use a standard chair-desk arrangement at will. (a) Weekly online performance surveys were administered to participants and their supervisors, as well as to all other sedentary employees and their supervisors. Using within-person statistical analyses, we find that overall work performance, quality and quantity of performance, and interactions with coworkers improved as a result of adoption of treadmill workstations. (b) Participants were outfitted with accelerometers at the start of the study. We find that daily total physical activity increased as a result of the adoption of treadmill workstations.

## Introduction

Sedentariness and physical inactivity cause or aggravate, for most people, a myriad physical illnesses [1], obesity [2] and psychological problems [3], [4] and reduce life expectancy [5]. These increase health care costs [6], [7] and reduce employee performance [8], [9]. Conversely, the effect of physical activity on health is positive; the greatest health improvements due to additional activity occur among individuals who have the lowest baseline levels of physical activity [10]. There is therefore a private and public interest in engaging greater numbers of people in physical activity. Alas, physical activity is not free: it frequently costs time and money, and for most people it is a source of direct disutility [11]. Because of a combination of ignorance, preferences, externalities and unrealistically high time-discount rates, most individuals engage in a level of physical activity below that deemed by many observers as individually and socially optimal.

Physical activity may be part of normal daily activities as a natural by-product of other activities and at no additional cost, such as physical work, walking to get to places and doing house chores, but familiar technologies have diminished substantially these activities [12]. One way to compensate for this trend is to reduce the relative price of physical activity. Providing incentives to exercise in the expectation of forming habits that allow for subsequent removal of the incentives is one way, but it appears to be effective only for few people [13]. Making it easier to walk and bike by creating special lanes or paths has had a small impact on physical activity [14].

Recent research has suggested that the decrease in physical activity at work may have been a more substantial contributor to the obesity epidemic than leisure time activities [15]. Some researchers have shown that simple interventions to increase activity at work – recommendations to walk stairs, stand up occasionally and walk during breaks – do result in small increases in physical activity [16], [1].

The effects of physical activity on employee performance are less clear-cut. No association between self-reported physical fitness and work performance was found in one study [17]. In another survey-based study, a positive association between physical activity and quality and quantity of performance was reported [18]. A review suggests that fitness intervention programs decrease sickness absence [19]. The first study that uses a within-person experimental design found that employees’ self-rated job performance and mood were higher on days they exercised in the company gym than on days they did not [20].

Since the lack of physical activity is closely associated with sedentariness at work [21], an obvious fix is to increase activity there. We conduct a workplace intervention, heeding the call of researchers to find practical interventions that involve the workplace [22]. We invite sedentary office workers to use treadmill workstations and measure their work performance and physical activity for up to one year.

### Conceptual framework and relevant literature

Workplace interventions intended to enhance fitness have been shown to increase physical activity and to reduce body fat [23], [24], [25], [26]. However, some studies fail to show that the intervention increases physical activity [27] and for most biometric health outcomes the evidence is less conclusive, if they are studied at all [23], [29] (for a disagreeing perspective, see [30]). Empirical studies in this area are generally difficult to interpret because they often lack randomization and longitudinal designs [31], [29]; recent studies incorporate these features and have more positive results [33].

We develop a conceptual framework that focuses on the effects of the introduction of treadmill workstations on physical activity and the performance of sedentary employees who type on a keyboard, speak on the phone, define problems and identify solutions to them and participate in meetings. Walking – when employees choose to walk instead of standing or sitting – while working entails a moderate physical effort and represents a completely new experience for most employees.

#### a. Effects on total physical activity (at and after work)

Consider an individual who allocates her daily time among sedentary, light and active physical activities. The allocation does not affect her short-term income, so it is determined by the individual’s preferences and the relative price of the three levels of activity to the individual. (Physical activity may affect long-term income through various channels, such as better health and improved performance; however, although everyone benefits on the long run from physical activity only few exercise.) These “prices” reflect ease of access, comfort level, social pressure to be active and ability to carry out physical activities.

The ready availability of a treadmill lowers the cost of engaging in physical activity because walking is concurrent with completing work tasks and its presence sends a reminder to engage in physical activity. Regular physical activity may be habit-forming, at least for a minority of participants [35], [13], so walking while working as well as after work may become easier after a while.

Inactive individuals have high – real or perceived – costs of exercise, so the introduction of treadmill workstations will be more effective for them than for others. Overweight and obese office workers who had treadmill workstations in their offices improved their waist and hip circumferences [43] and lost weight [44]. On the other hand, already-active individuals may regard walking while working a substitute for exercise; the net effect on total physical activity depends on the size of the substitution effect.

Volunteering to participate in the study may act as a self-commitment device to exercise. Furthermore, in our study the company provided treadmill workstations and reconfigured their offices without requiring participating employees to use them for a particular length of time; some may reciprocate the trust placed in them by using the equipment [41].

In sum, we expect that the changes in relative prices and preferences will induce an increase in physical activities of sedentary individuals. We therefore hypothesize:

*H1. The introduction of treadmill workstations enhances users’ overall physical activity.*

#### b. Effects on work performance

The introduction of treadmill workstations may impact performance via health improvements and the ability to handle stress [40]. Indeed, physical and emotional well-being enhances job performance [40]. The treadmill workstations may also impact work performance in other ways. First, the treadmill work stations represent a gift from the employer to participating employees who may reciprocate the employer’s unconditional gift by working better and harder and shirking less, at least for a while [41]. Second, working – typing, writing, reading, speaking and thinking – while walking is an instance of multitasking. Walking and work tasks may complement or compete with each other. Walking is a hindrance to tasks that require a steady posture and the use of hands for precise execution, such as typing and using a computer mouse [36], [45]. On the other hand, walking may complement the execution of complex cognitive tasks [37] because it reduces stress, increases the size of the hippocampus and improves memory [38], and helps with focus and concentration on cognitive tasks. However, a study on performance of simulated office work tasks carried out on a treadmill workstation found that, during the two-day study, math scores were lower and selective attention and processing and reading comprehension were no different from a seated position [43]. It is not clear whether performance would have improved if these tasks were familiar and frequently repeated, as is the case in actual office environments.

The implementation of treadmill workstations does not have to be rigid. In the present study, employees have discretion to adjust the treadmill speed as they see fit, from 0 mph (standing or sitting) to 2 mph. Thus employees can optimize the speed relative to the task at hand, for example standing or sitting when typing, walking very slowly when talking on the phone and taking hand-written notes, and walking faster when thinking about complex problems. Employees may require some time to learn how best to carry out their various work tasks in combination with walking on the treadmill, during which performance may well decline. Subsequently performance will rise again and exceed the performance level before the introduction of the treadmills. It is difficult to predict the duration of the learning period and transitioning from a life-long desk-and-chair way of working to a partly walking, partly standing and partly sitting way of working.

On the basis of the discussion above we formulate our key performance-related hypothesis:

*H2. The introduction of treadmill workstations enhances users’ work performance once they learn how to adjust to working effectively in the new environment.*

## Methods

### Ethics statement

The experiment was approved by the Mayo Clinic’s institutional review board, and volunteers provided written consent. The survey portion was approved by the University of Minnesota’s institutional review board, and participants provided electronic consent before they completed their first survey. The consent form protected the privacy of the participants, reading “Only your ID will be on the survey; researchers will not have access to your name or contact information. Your employer will not have access to your surveys. Research records will be stored securely, and only researchers will have access to these records.” The data, with identifying information removed, are available to researchers for replication purposes. Please contact Prof. Darla Hamann.

### Design

**Figure 1:**
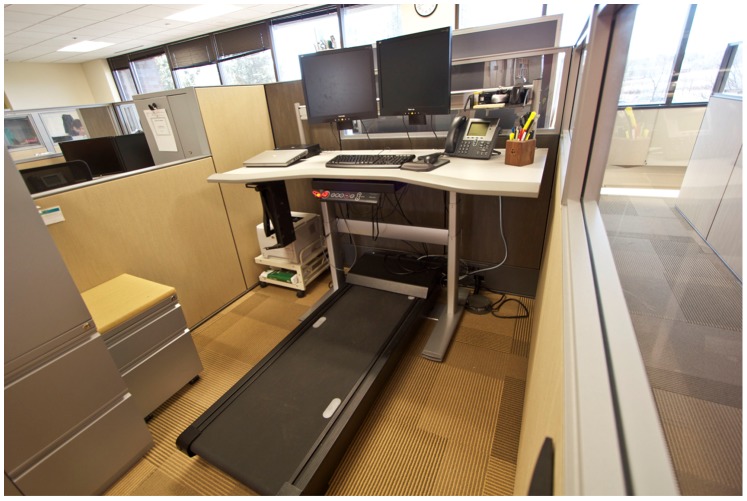
Treadmill workstation image.

A national financial services company headquartered in the Twin Cities agreed to be the site of the experiment and funded it. Experiment participants’ existing standard offices were refitted such that a computer, phone and writing space were placed on a desk in front of a treadmill operated by the employee at speeds between 0 and 2 mph. The desk can be lowered by the press of a button that activates a hydraulic motor with the treadmill becoming a stable platform for a chair. The treadmill desk (see Figure 1) was made by Steelcase, Grand Rapids, Michigan. It does not record any measure of usage. No relocation was necessary.

An email invitation to participate in the experiment was issued by the company to 409 employees whose jobs were sedentary. The company made it clear that participation in the study was voluntary, that there was no expectation that participants walk a certain amount and that the primary concern was the health of the employees. When 43 employees (who were not pregnant nor advised by their physician to refrain from participation in the experiment) volunteered, enrollment into the study was closed, and the first 40 volunteers were randomly assigned to one of two groups with 20 participants in each; the remaining three volunteers were wait-listed. Members of one group received treadmills in June 2008 and are referred heretofore as Walker 1. Members of the other group received treadmills in late December 2008 and are referred to as Walker 2. The experiment ended as planned after 12 months, at the end of May 2009. Four Walker 1 participants dropped out from the study: one because of the diagnosis of inflammatory bowel disease, one because of pregnancy, one developed connective tissue disease requiring high dose steroid use and one left the company. The wait-listed volunteers were added to Walker 2. The remaining treadmill workstation was given to an employee in week 16 when the first Walker 1 volunteer dropped out, but this employee was not given an accelerometer at this late date. The final sample consisted of 17 Walker 1 and 23 Walker 2 who completed surveys, although not everyone completed the survey each week. Usable energy expenditure data are available for 16 Walker 1 and 23 Walker 2. Company employees who did not receive treadmills constitute the Non-Walker group who participated, along with Walker 1 and Walker 2, in the weekly survey portion of the study.

The three groups are quite similar. Most are female (73% of Non-Walker, 67% of Walker 1 and 81% of Walker 2), a large minority has college education (31% of Non-Walker, 44% of Walker 1 and 29% of Walker 2), most are married (67% of Non-Walker and 61% of both Walker 1 and Walker 2), and they spend most of the working day on the computer (6.44 hours for Non-Walker, 6.07 for Walker 1 and 5.93 for Walker 2), working on moderately complex tasks (3.5 for Non-Walker and Walker 1 and 3.3 for Walker 2, on a 1–5 scale). Additional information concerning differences between Non-Walker and Walker 1 and 2 is presented in the Conclusions.

#### a. Data collection

Data on performance and work-related activities and events were collected through surveys and from company administrative records. Walker 1, Walker 2 and Non-Walker received an online detailed quarterly questionnaire concerning work, life and health. In addition, a three-minute survey was administered online every Wednesday. The company encouraged all employees to fill out the surveys on company time. Supervisors received surveys focusing on each of their supervisees, concentrating on key questions that paralleled the work-related questions asked in the employee surveys. Each supervisor has on average 10 supervisees. Supervisors filled out surveys also as employees. We administered the weekly surveys 50 times and the quarterly surveys four times. Changes in the company workforce – separations, hires, moves within the company and promotions to supervisory roles – were reported to us immediately and were reflected in the type of survey affected employees received and were accounted for in our analyses. The overall weekly employee survey response rates averaged 39%, whereas for participants in the study, the Walkers, it was 68%; for supervisors it averaged 41%.

**Figure 2:**
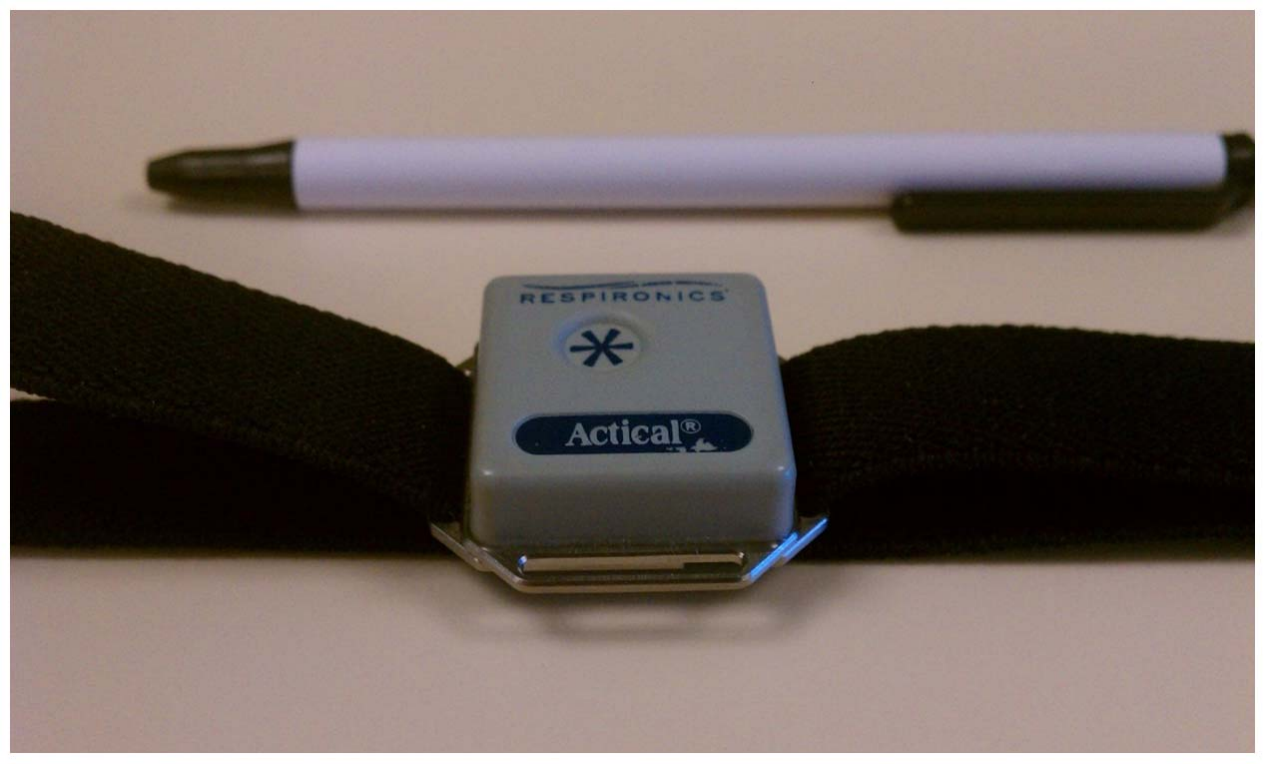
Accelerometer image.

About a month before Walker 1 were provided treadmill desks, both they and Walker 2 were outfitted with accelerometers (see Figure 2), energy expenditure monitoring devices that were worn continuously during waking hours. The device (Actical; Respironics, Philips, Eindhoven, The Netherlands) measures physical activity by recording activity (accelerations gathered at 32 hertz, stored on the device internal memory). Very little non-wear time was observed.

#### b. Measures

*Physical activity of participants in the experiment* (Walker 1 and Walker 2) is captured by two variables derived from the measurements obtained through the accelerometer. One variable is the *total daily activity caloric expenditure,* averaged over a week; the conversion of accelerometer data into caloric expenditure is described in [44]. We measure activity over the entire day because the company does not have fixed working hours, and because we want to capture the effect of treadmill workstations on combined physical activity, at work and outside work. The second dependent variable concerns *the allocation of time (in minutes) among different levels of intensity*: *sedentary*, equivalent to sitting or walking at a speed of less than 1 mph, *light*, equivalent to a speed of 1–2 mph, and *active,* equivalent to a speed higher than 2 mph. The duration of use of the treadmill was not recorded. *Employee performance* is captured by several variables.

Overall performance was assessed for the week preceding the survey “On a scale from 0 to 10 where 0 is the worst job performance anyone could have at your job and 10 is the performance of a top worker, how would you rate your usual job performance during the past week?” We also asked about the employee’s quality of performance (average of “Consider your work yesterday, Tuesday. Please rate the quality of your work” and “Now consider the day before that, Monday. Please rate the quality of your work.” Scored from 1 (Poor) to 5 (Far above average)), quantity of performance (average of “Consider your work yesterday, Tuesday. Please rate the quantity of your work” and “Now consider the day before that, Monday. Please rate the quantity of your work.” Scored from 1 (Poor) to 5 (Far above average)) and quality of interactions with coworkers for the day before the survey (a Tuesday) and for the day before that (a Monday) (average of “Consider your work yesterday, Tuesday. Please rate the quantity of your work” and “Now consider the day before that, Monday. Please rate the quantity of your work.” Scored from 1 (Poor) to 5 (Far above average)). Asking for four different dimensions of performance and for slightly different periods provides a more complete picture of an employee’s assessment of his or her own performance than would be afforded by a single item and a single time frame. The measures are similar to those employed by [18], and were discussed with the company’s management, who agreed that they capture critical dimensions of work performance that are used for performance evaluation and are comparable over time and across jobs. Supervisors were asked to rate their ten employees (on average) on the same four dimensions using items that were nearly identical to those asked of employees. Each supervisor had to complete a survey as an employee.

##### Treadmill workstation

The main independent variable is the availability of a treadmill workstation to a participant in a particular week, an indicator variable taking the value 1 if the employee had a treadmill and 0 otherwise. To identify the role of experience and learning over time we use the number of weeks that a participant had a treadmill workstation, as suggested by [42].

##### Control variables

Additional factors may influence physical activity and performance. We include illness (days absent from work due to illness during the week prior to the weekly survey), the move of the company to a different location (staggered over a period of weeks) and change in work duties.

#### c. Analysis

The analysis identifies the effect of treadmill workstations and experience with them on the basis of within-person changes in physical activity and work performance of Walkers (and in the case of performance, using Non-Walkers information to account for company-wide trends). In the estimation of the panel data (caloric expenditure and performance) we use generalized least squares regression (GLS). We performed standard econometric tests for serial correlation, finding that we could not reject the null hypothesis of no serial correlation for most models. The appropriate analytical method is therefore the AR(1) auto-regressive error correction that accounts for correlations between the error terms of two consecutive weeks. The tests are described by [46]. For estimation we use Stata procedure *xtregar,* which can accommodate unbalanced panels with observations that are unequally spaced over time. It implements the methods derived by [47]. In the estimation of caloric expenditure, where only Walker 1 and Walker 2 data are used, we account for possible unobserved heterogeneity by using a random effects model. The preferred method, fixed effects estimation, reduces the degrees of freedom and produces similar but somewhat larger estimates. In the estimation of performance, where we have data also for Non-Walker employees, we use fixed effects models. In the estimation of the allocation of time among three levels of activity intensity we estimate a system of three interdependent equations, using seemingly unrelated regression (SUR) to account for the correlation of the error terms across the equations, as well as a time trend to account for correlation of error terms over time.

We present two models: Model 1 includes only the treadmill dummy variable, whereas Model 2 includes also the number of weeks the employee had a treadmill up to the current week and the square of the number of weeks to detect a possible nonlinear relationship between activity and time. We treat the dependent variable, an ordinal construct, as if it were cardinal and use relevant estimation methods described in the text. Like many other researchers, we find that ordered logit, the proper method for ordered data, gives similar results (not reported here), essentially because the minimum and maximum values are rarely invoked, hence there is no potential censoring problem.

## Results

Tables 1 and 2 present the variables used in the analyses, their sources, and descriptive statistics. Table 1 focuses on physical activity and Table 2 on work performance, separately for the first 29 weeks, when only Walker 1 received treadmills, and for the subsequent 23 weeks, when both groups had treadmills. For the first 29 weeks for Walker 1, the panel includes only observations when they had treadmills (which they received in a staggered fashion), so that the figures can be compared with those of Walker 2, who did not have treadmills during this period. Walker 1 spent 1,200.3±27.2 daily calories as compared to Walker 2′s 896±16; this is possibly a consequence of the use of treadmills by those who had them (Walker 1), but may also be due to random differences in activity level between members of the two small groups. The allocation of time across different levels of activity reflects the difference in caloric expenditure. Walker 1 spent more time being active (110.7±5.47 minutes) and less time being sedentary (969.1±10.47 minutes) as compared to Walker 2 (47.5±2.4 active and 1082.9±5.76 sedentary minutes). The between-group differences evaluated with the Mann-Whitney-Wilcoxon (MWW) nonparametric test are statistically significant at the.05 alpha level. The average total daily caloric expenditure for Walkers 1 and 2 in the second period, when they both had treadmills, shows a small decline as compared to Walker 1 in the first period but an increase relative to Walker 2. The first period includes summer and fall, which present more opportunities for outdoor activities than the second period, which includes winter and spring. Of course, in such a small sample these between-person estimates of calories burned could be attributable to individual factors, so we need to rely on a multivariate analysis to control for individual heterogeneity.

**Table 1:** Definition of Variables, Sources and Descriptive Statistics: Physical Activity Variables, Walkers Only.

Variable	Definition	Source	Mean During First 29Weeks ± Std Error			Mean During Weeks30–52± Std Error
			*Walker 1*		*Walker 2*	*All Walkers*
Activity caloric expenditure	Total activity calories per day	Accelerometer	1200.3±27.2		896.0±16.1	989.0±15.67
Active minutes	Daily minutes of energyexpenditure equivalent towalking >2 mph	Accelerometer	110.7±5.47		47.5±2.43	77.8±3.09
Light minutes	Daily minutes of energyexpenditure equivalent towalking 1–2 mph	Accelerometer	358.9±8.77		309.6±5.17	304.2±4.63
Sedentary minutes	Daily minutes of energyexpenditure equivalent towalking <1 mph	Accelerometer	969.1±10.47		1082.9±5.76	1052.7±5.76
Had treadmill duringthe current week	Employee has a treadmillin the office during thecurrent week (dummy)	AdministrativeData		0.79±0.02	0	1
Number of weekswith treadmill	Number of weeks employeehas had treadmill up to present	AdministrativeData		9.15±0.35	0	21.61±0.049

**Notes:** Walker 1 received treadmills in a staggered fashion, usually between weeks 5 and 8, and Walker 2 between weeks 30–34. Weeks for which Walkers did not have treadmills are excluded.

**Table 2:** Definition of Variables, Sources and Descriptive Statistics: Employee Performance and Work-Related Variables, All Survey Participants.

Variable	Definition	Source	Mean DuringWeeks 1–29± Std Error			Mean During Weeks30–52± Std Error	
*Employee Performance Measures – Employee self-reports*			*Walker 1*	*Walker 2*	*Non-Walker*	*Walkers*	*Non-Walkers*
Overallperformance	Past week’s overallperformance	EmployeeWeekly Survey	7.37±0.09	6.98±0.08	7.81±0.02	7.29±0.07	7.83±0.03
Performancequantity	Past two days’ quantityof work done	EmployeeWeekly Survey	3.44±0.04	3.33±0.04	3.61±0.01	3.55±0.03	3.68±0.02
Performancequality	Past two days’ qualityof work done	EmployeeWeekly Survey	3.51±0.04	3.37±0.03	3.70±0.01	3.52±0.03	3.76±0.01
Interactionquality	Past two days’ quality ofinteraction with coworkers	EmployeeWeekly Survey	3.62±0.04	3.29±0.03	3.49±0.02	3.50±0.03	3.56±0.02
*Employee Performance Measures – Supervisor reports*							
Overallperformance	Past week’s overallperformance	SupervisorWeekly Survey	6.42±0.10	7.32±0.08	6.95±0.02	6.78±0.08	7.24±0.03
Performancequantity	Past two days’ quantityof work done	SupervisorWeekly Survey	3.24±0.05	3.59±0.05	3.51±0.01	3.45±0.04	3.64±0.02
Performancequality	Past two days’ qualityof work done	SupervisorWeekly Survey	3.26±0.05	3.56±0.03	3.56±0.01	3.51±0.04	3.53±0.01
Interactionquality	Past two days’ qualityof interaction with coworkers	SupervisorWeekly Survey	3.37±0.05	3.42±0.03	3.47±0.01	3.43±0.04	3.47±0.01
Days absentdue to illness	Days absent from workdue to own illness duringthe past week	EmployeeWeekly Survey	0.07±0.02	0.02±0.01	0.08±0.01	0.08±0.02	0.12±0.01
Moved officelocation	Packed or moved to anew location duringthis week = 1	AdministrativeData	0.02±0.01	0.02±0.01	0.02±0.00	0.03±0.01	0.01±0.00
Changedduties	Change in employeeduties or responsibilities	Administrativeand Survey Data	0.10±0.01	0.04±0.01	0.03±0.01	0.05±0.01	0.02±0.00

Note: Walker 1 received treadmills in a staggered fashion, usually between weeks 5 and 8, and Walker 2 between weeks 30–34. Weeks for which Walkers did not have treadmills are excluded.

Turning to performance measures in Table 2, the grand mean of overall self-rated Walker 1 performance during the first 29 weeks of the study was 7.37±0.09, that of Walker 2 it was 6.98±0.08 and of Non-Walker it was 7.81±0.02. The supervisor-rated comparable figures are 6.42±0.1, 7.32±0.08 and 6.95±0.02, respectively. The comparative figures for quality, quantity and interaction quality of performance are similar. Using the MWW nonparametric tests, we find that all of the differences are statistically significant, with the exceptions of self-rated performance quality, self-rated performance quantity, and supervisor-reported interaction quality. Whether any differences between Walker 1 and Walker 2, and between Walker 1 and Non-Walker, are due to the use of treadmills by Walker 1 is impossible to assess on the basis of these figures and tests. First, the performance levels of Walker 1 and Walker 2 may reflect differences in performance levels unrelated to and predating the study. For example, considering the five weeks prior to when Walker 1 began receiving treadmills, employee overall self-rated performance was 7.78±0.12 for Walker 1 and 7.30±0.15 for Walker 2 (statistically significant at the 0.10 alpha level using MWW tests), and supervisor-rated performance was 6.92±0.19 for Walker 1 and 7.62±0.19 for Walker 2 (statistically significant at the 0.01 level using MWW tests). Second, performance averaged over many weeks may conceal nonlinear changes during the period under consideration. Changes from the first 29 weeks to the subsequent 23 weeks in average performance over the diverse measures and across the Walker and Non-Walker groups exhibit a mixed pattern, with Walkers’ second period mean lying between the means of Walker 1 and Walker 2 in the first period, and for Non-Walkers generally registering little change. But this comparison over time can shed no light on the effect of the availability of treadmill workstations on performance, and we need to proceed to an analysis that takes into account heterogeneity, possible nonlinearity of effects over time, and other factors that may influence employee performance in the context of a within-person trend analysis.

At the bottom of Table 2 we report descriptive statistics for control variables.

### 1. Physical activity

In Table 3 we examine total activity calories expended on an average day each week. The overall effect of the treadmill dummy in both models is about 74 additional calories a day (p<0.01). This amounts to an increment of around 7–8% in daily activity caloric expenditure. Model 2 suggests that the activity level associated with the treadmill is concave in time, reaching a peak after about 10 weeks and adjusting down afterwards. In Table 4 we examine caloric expenditure by time of day: day, 7 AM–5 PM, which corresponds to common working hours (but recall that our company does not have fixed working hours), evening, 5 PM–11 PM and night, 11 PM–7 AM. Having a treadmill in the office is associated with a relatively large increase in caloric expenditure during the day (β = 60.5, p<0.05) and a smaller (β = 21.2, p<0.01) increase in the evening, and none at night (β = −2.36, p>0.10).

**Table 3:** Average Daily Activity Caloric Expenditure for Walkers–Random Effects Generalized Least Squares with AR(1) Errors.

	Model 1	Model 2
Had treadmill duringthe current week	74.4±30.9***	73.0±39.5*
Number of weekswith treadmill		4.25±4.38
(Number of weekswith treadmill)^2^		−0.21±0.10**
Absence due to illness	−6.33±23.41	−6.97±23.3
Moved office locations	−18.96±49.4	−19.4±49.2
Changed duties	41.4±40.7	34.7±40.8
Constant	959.6±65.7***	971.5±62.3***
Number of observations	1173	1173
Wald chi^2^	6.99	20.14
Prob>chi^2^	0.2214	0.0053

**Notes:** (1) Standard errors are corrected for serial correlation. (2) *, ** and *** indicate statistical significance at the 0.1, 0.05 and 0.01 levels, respectively. (3) “Had treadmill during the current week” is an indicator variable talking on the value of 1 when the walker had a treadmill in his or her office, and 0 when he or she did not have a treadmill in his or her office.

**Table 4:** Activity Caloric Expenditure by Time of Day – Replication of Table 2, Model 2: Random Effects Generalized Least Squares with AR(1) Errors.

	Day	Evening	Night
Had treadmill during the current week	60.5±30.9**	21.2±8.08***	−2.36±4.60
Number of weeks with treadmill	4.39±3.46	−1.07±0.90	0.29±0.51
(Number of weeks with treadmill)^2^	−0.17±0.08**	−0.02±0.02	−0.01±0.01
Absence due to illness	−5.45±17.83	1.21±4.95	−0.74±2.81
Moved office locations	−18.49±37.4	4.77±10.26	−5.77±5.83
Changed duties	13.83±28.5	6.70±7.99	0.71±4.53
Constant	736.9±43.8***	151.4±14.4***	67.13±8.14***
Number of observations	1213	1219	1219
Wald chi^2^	19.40	36.89	1.51
Prob>chi^2^	0.0070	0.00	0.9821

**Notes:** (1) Standard errors corrected for serial correlation. (2) *, ** and *** indicate statistical significance at the 0.1, 0.05 and 0.01. (3) “Had treadmill during the current week” is an indicator variable talking on the value of 1 when the walker had a treadmill in his or her office, and 0 when he or she did not have a treadmill in his or her office. (4) Day: 7 AM –5 PM, Evening: 5 PM –11 PM, Night: 11 PM –7 AM.

In Table 5 we investigate the allocation of time among three levels of intensity of physical activity – sedentary, light and active. We use seemingly unrelated regression (maximum likelihood estimation with robust standard errors) to account for the interdependence among the three activity levels. We use the *mysureg* procedure in Stata 12, provided by [48]. Models 1 and 2 are similar to those in Table 3.

**Table 5:** Average Daily Allocation of Time among Different Activity Intensity Levels for Walkers – Seemingly Unrelated Regression with Robust Standard Errors.

	Minutes Spent in:					
	*Sedentary (<1 mph)*		*Light (1–2 mph)*		*Active (>2 mph)*	
	Model 1	Model 2	Model 1	Model 2	Model 1	Model 2
Had treadmill during the current week	−77.46±11.7***	−60.48±13.57***	40.82±9.9***	46.75±11.2***	38.90±7.1***	11.90±8.7
Number of weeks with treadmill		−2.33±1.53		−0.91±1.24		4.31±0.95***
(Number of weeks with treadmill)^2^		0.02±0.04		0.02±0.03		−0.07±0.02***
Absence due to illness	2.88±12.8	2.00±12.9	3.43±12.4	3.34±12.5	−8.60±4.0**	−8.60±4.37**
Moved office locations	20.7±22.2	21.4±22.3	−28.8±18.4	−28.7±18.2	7.1±17.0	6.79±16.7
Changed duties	−13.9±16.7	−11.9±16.4	−42.7±10.3***	−42.4±10.4***	58.2±10.6***	57.6±10.1***
Constant	1120.9±14.6***	1118.0±14.4***	279.4±12.3***	278.3±12.15***	33.7±5.9***	39.15±5.78***
Number of observations	1220	1220	1220	1220	1220	1220
Wald chi^2^	199.9	239.5	199.9	239.5	199.9	239.5
P-value	0.000	0.000	0.000	0.000	0.000	0.000

**Notes:** (1) Estimations include a time trend and its square. (2) Standard errors are robust. (3) *, ** and *** indicate statistical significance at the 0.1, 0.05 and 0.01 levels, respectively. (4) “Had treadmill during the current week” is an indicator variable talking on the value of 1 when the walker had a treadmill in his or her office, and 0 when he or she did not have a treadmill in his or her office.

The results suggest that the availability of a treadmill to a participant is associated with reallocation of time across the three levels of activities, away from sedentary to light and active activities. The point estimate of having a treadmill workstation in Model 1 is about 77 fewer sedentary minutes per day (p<0.01). This number should be compared to the average daily sedentary time of approximately 1,173 minutes (estimated constant). Approximately 500 minutes of sedentary time may be accounted for by sleep and another 50 minutes for commuting to work. (These are approximate values derived from *America Time Use Survey 2009*, U.S. Bureau of Labor Statistics.) This leaves about 600 ‘discretionary’ minutes for non-sedentary activity, which includes approximately 500 minutes at work during a weekday. Light activities increase by about 41 minutes per day (p<0.01); (compare with 279 minutes, the estimated constant) and active minutes by about 39 minutes (p<0.01) (compare with 34 minutes, the estimated constant). The two add up to 80 rather than 77; the small discrepancy arises because these point estimates were not constrained to add up to zero. Model 2 indicates that time spent in active physical activity is increasing over time; the rate of increase declines slowly over time (reaching an estimated maximum at week 307, quite outside our sample range). There is commensurate convexity, imprecisely measured (p>0.10), in sedentary and light activities (with estimated minima at weeks 35 and 64, respectively). Our results support Hypothesis 1: daily physical activity increases with the introduction of treadmill workstations.

### 2. Work performance

To evaluate changes in employee performance over time and to test Hypothesis 2 we examine employees’ weekly self-ratings as well as supervisor weekly rating, pooling data for Walker 1, Walker 2 and Non-Walker. There are more than 7,000 employee weekly observations from employee reports but less than 4,000 observations from supervisors. The discrepancy arises from the fact that we use supervisor reports only for weeks when their employees also completed the weekly survey (we did not eliminate observations for employees for weeks that their supervisors did not complete their surveys). Eliminating this constraint does not affect the results. Table 6 presents results for overall performance (on a scale of 1–10). Model 1 suggests that the availability of a treadmill workstation is associated with a 0.69 points (p<0.01) increase in employee self-rated overall performance (the grand mean is around 7.5) and 1.11 points (p<0.01) increase in supervisor-rated overall performance (the grand mean is about 7.0). Both self-and supervisor-rated performance declines first and increases subsequently, according to Model 2 (p<0.01 for employee-rated performance, p>0.10 for supervisor-rated performance). The point estimates for the employee self-rating imply that performance bottoms out after almost 24 weeks and then starts rising again. The supervisor-rated performance, imprecisely estimated (p>0.10), bottoms out after almost 21 weeks, but these estimates are not statistically significant. Note that the estimation in Table 6 as well as in Table 7 includes Non-Walkers, whose performance information is used to capture any company-wide trends in performance. If we exclude Non-Walkers from the estimation, the results in Table 6 as well as in Table 7 remain essentially unchanged.

**Table 6:** Weekly Overall Performance for All Survey Respondents–Fixed Effects (Within-Person) Generalized Least Squares Regression with AR(1) Errors.

	*Employee-Rated Performance*		*Supervisor-Rated Performance*	
	Model 1	Model 2	Model 1	Model 2
Had treadmill during the current week	0.69±0.11***	0.90±0.13***	1.11±0.16***	1.16±0.20***
Number of weeks with treadmill		−0.04±0.02***		−0.01±0.02
(Number of weeks with treadmill)^2^ * 10^3^		0.76±0.31***		0.29±0.48
Moved office locations	0.36±0.08***	0.36±0.10***	0.34±0.10***	0.35±0.10***
Absence due to illness	−0.17±0.03***	−0.17±0.03***	0.06±0.05	0.06±0.05
Changed duties	0.03±0.05	0.03±0.05	0.20±0.07***	0.20±0.07***
Constant	8.11±0.01***	8.12±0.02***	7.95±0.02***	7.95±0.02***
N	7325	7325	3679	3679
F	23.6	16.87	17.81	12.60
Prob>F	0.00	0.00	0.00	0.00

**Notes:** (1) Standard errors are corrected for serial correlation. (2) *, ** and *** indicate statistical significance at the 0.1, 0.05 and 0.01. (3) “Had treadmill during the current week” is an indicator variable talking on the value of 1 when the walker had a treadmill in his or her office, and 0 when he or she did not have a treadmill in his or her office.

**Table 7:** Determinants of Different Dimensions of Weekly Performance – Fixed Effects (Within-Person) Generalized Least Squares Regression with AR(1) Errors.

	*Employee Rated Performance*			*Supervisor Rated Performance*		
	Quantity	Quality	Interaction	Quantity	Quality	Interaction
Had treadmill during the current week	0.40±0.08***	0.39±0.07***	0.39±0.07***	0.62±0.11***	0.62±0.11***	0.57±0.10***
Number of weeks with treadmill	−0.01±0.01	−0.02±0.01***	−0.02±0.01**	−0.01±0.01	−0.00±0.01	−0.00±0.01
(Number of weeks with treadmill)^2^ * 10^3^	0.30±0.18*	0.44±0.12***	0.33±0.17**	0.24±0.27	0.11±0.26	−0.00±0.00
Absence due to illness	0.04±0.02**	0.04±0.02**	0.04±0.02**	0.09±0.03***	0.11±0.03***	0.07±0.03**
Moved office location	0.14±0.05***	0.15±0.04***	0.17±0.04***	0.10±0.06*	015±0.06***	0.19±0.06***
Changed duties	0.21±0.03***	0.13±0.02***	0.15±0.03***	0.15±0.04***	0.10±0.04**	0.14±0.04***
Constant	3.81±0.01***	3.89±0.01***	3.71±0.03***	4.02±0.01***	4.080±0.01***	3.92±0.01***
N	7054	7056	7054	3477	3477	3477
F	15.47	12.23	13.85	9.76	12.86	12.98
Prob>F	0.000	0.000	0.000	0.000	0.000	0.000

**Notes:** (1) Standard errors are corrected for serial correlation. (2) *, ** and *** indicate statistical significance at the 0.1, 0.05 and 0.01. (3) “Had treadmill during the current week” is an indicator variable talking on the value of 1 when the walker had a treadmill in his or her office, and 0 when he or she did not have a treadmill in his or her office.

**Figure 3:**
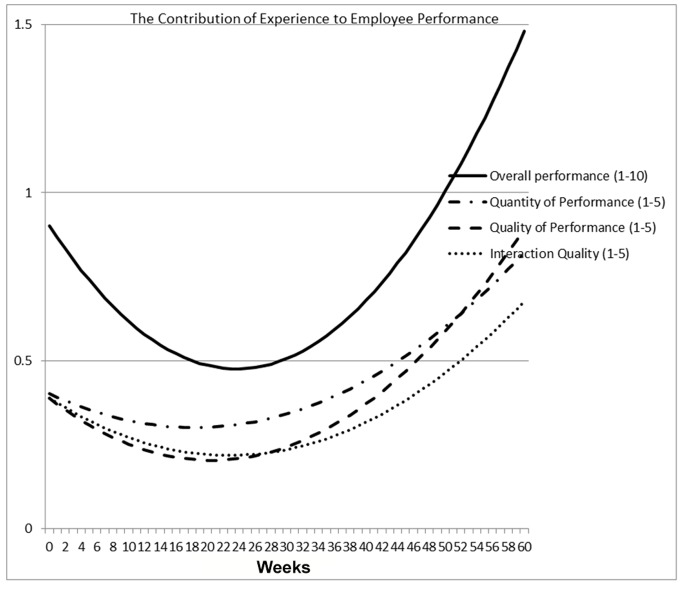
The Treadmill Workstation Learning Curve - The Contribution of Experience to Employee Performance.

Table 7 presents results from similar models for the quantity of performance, quality of performance and the quality of interaction with co-workers, each on a scale of 1–5 (overall performance was on a 1–10 scale). The weekly survey question on overall performance refers to the previous week, whereas the questions about quality, quantity and interaction with coworkers refer to Monday and Tuesday prior to the weekly survey, which was administered on Wednesday. If the employee was absent (for any reason) these days the observation was recorded as missing. As a result, we have slightly more observations for Table 6 than for Table 7. For conciseness we report results for Model 2 only. For all performance sub-dimensions, performance is higher when the treadmill is present in an employee’s office than when it is not (p<0.01). Ratings of sub-dimensions of performance follow the same pattern as for overall performance, with an initial decline followed by a subsequent increase, and with the time pattern imprecisely estimated for supervisors (p<0.01 for employee-rated performance quality and interaction quality, p>0.10 for supervisor-rated performance sub-dimensions). Quantity of performance bottoms out at approximately week 18 for employee self-rating and almost week 23 for supervisor-rating, quality of performance at week 20 and 14 for employee and supervisor ratings respectively, and quality of interaction with co-workers at week 23 for self-rating and flat for supervisor rating. The estimated turnaround point occurs a few weeks earlier for sub-dimensions of performance than for overall performance, but the pattern is similar and the discrepancy is not large. Figure 3 illustrates this graphically; it plots the fitted relationship between experience with treadmill workstations and the employee self-rated performance associated with treadmills, using the point estimates for the treadmill variables from Tables 6 and 7 (extrapolating beyond the sample period to 60 weeks).

## Conclusions

The results suggest that the introduction of treadmill workstations, as hypothesized, has a significantly favorable impact on both physical activity and work performance. The total average daily activity caloric expenditure of participants increased by more than 74 calories, the consequence of a decline of more than an hour a day in sedentary activities and a concomitant increase in light and active activities. This effect is generated over the year-long duration of our experiment. Walking at work does not seem to have come at the expense of much, if any, physical activity after work; the activity effects we measured are over the entire day.

Did participants trade off work for walking on the treadmill? For the entire year-long period the net performance effect of treadmill workstations is positive, amounting to about 0.69 points for employee self-rating and 1.11 for supervisor rating on a 1–10 scale. (It should be noted, however, that supervisor ratings are likely to be less sensitive to weekly changes in the performance of all of their supervisees, which may explain the lesser precision of estimates based on their ratings as compared to those based on employees’ own ratings). While we cannot determine the precise behavioral source of the performance improvements, our data are consistent with the favorable effect of physical activity on performance found by other researchers [18] using a within-person design.

The transition to the new work environment is not immediate; in fact, there is an early decline in performance while participants learn how to adjust to walking while working on various tasks. Our study suggests that it is important to examine nonlinear effects over a relatively long period of time. Had we ignored nonlinearity or considered only discrete changes over arbitrary periods, we would have not estimated correctly the effects of treadmill workstations on physical activity and work performance. Training in the use of treadmills for different tasks may shorten the adjustment and learning period, thus enhancing the positive effect of treadmill workstations.

The physical activity and performance gains can be contrasted with the cost of a treadmill workstation, about $4,000 in this experiment and around $1,000 on Amazon.com. It seems that companies ought to consider making treadmill workstations available to their sedentary employees. However, there are several limitations to our study that may restrict their applicability to other situations. The volunteers in our study were under the attention of researchers for an entire year, and their workstations looked different from those of their non-participating coworkers. Whether this has affected their behavior (walking more and working harder to justify their participation in the study) cannot be discerned from our study.

Furthermore, volunteers have self-selected into the experiment, and therefore they may have walked more and worked better than other employees would have if assigned to treadmill workstations. We examined whether participants differ systematically from the rest of the company’s workforce but found few significant or meaningful differences between the two groups. We ran a logit regression with participation in the study as the dependent variable, and baseline (before the experiment began) independent variables: age, gender, education, Body Mass Index (BMI), marital status, work hours, hours of computer use, job task characteristics (routine, complexity, decision-making, teamwork), health perceptions and actions (diet, health behaviors, exercise), and time use (sports and exercise and various activities). Participants were more likely to perceive themselves as overweight; however, their BMIs, calculated on the basis of their self-reported weight and height, did not differ significantly from other survey respondents. They were more likely to be younger, better educated and less likely to work in a team. In most other ways, however, the sample of volunteers looks similar to the other survey respondents in this company. We did not find any significant effect of hours of work, computer work, task characteristics, health behaviors or time use on the choice to participate. But the fact that only about 10% of employees chose to volunteer suggests the possibility of unobserved factors that distinguishes between the two groups. Volunteers were probably more motivated to increase their physical activity at work and outside work than other employees, which is what must have moved them to take up the opportunity to use treadmill workstations. However, while motivation to increase physical activity might have been present prior to our study, it was the opportunity to use treadmill workstations that allowed participants to act on this motivation and to increase the level of their physical activity.

Future research should address the effects of various contingencies that may affect the impact of treadmill workstations on physical activity and work performance. Important contingencies include the fitness level of employees and the nature of their tasks; it is possible that less physically fit employees and employees whose tasks are more cognitively complex will gain relatively more from the use of treadmill workstations. Future research should also investigate the specific behavioral channels through which working while walking affects work performance.
